# Hemophagocytic Lymphohistiocytosis Associated with Immune Checkpoint Inhibitors: A Pharmacovigilance Analysis of Spontaneous Reports

**DOI:** 10.3390/cancers18071164

**Published:** 2026-04-04

**Authors:** Suleyman Sami Guzel, Kubilay Tay, Ebru Cicek, Seda Jeral Evinc, Suheyla Atak, Cigdem Papila, Nebi Serkan Demirci, Ozkan Alan

**Affiliations:** Division of Medical Oncology, Department of Internal Medicine, Cerrahpasa Faculty of Medicine, Istanbul University-Cerrahpasa, Istanbul 34098, Turkey; suleymanguzel34@hotmail.com (S.S.G.);

**Keywords:** hemophagocytic lymphohistiocytosis, immune checkpoint inhibitors, FAERS

## Abstract

**Objectives:** Hemophagocytic lymphohistiocytosis (HLH) is a rare but life-threatening hyperinflammatory syndrome increasingly reported with immune checkpoint inhibitors (ICIs). However, comparative real-world data across different ICI classes and treatment strategies are limited. This study aimed to characterize HLH reporting patterns associated with ICIs and to compare disproportionality signals among PD-1 inhibitors, PD-L1 inhibitors, and combination regimens using the U.S. Food and Drug Administration Adverse Event Reporting System (FAERS). **Methods:** A retrospective pharmacovigilance analysis was performed using FAERS reports submitted between 2013 and 2025. HLH-related cases were identified using core Medical Dictionary for Regulatory Activities (MedDRA) Preferred Terms. Reporting odds ratios (RORs) with 95% confidence intervals (Cis) were calculated to assess disproportionality across ICI treatment strategies, with ICI monotherapy as the reference. Restricted analyses compared PD-1 inhibitors, PD-L1 inhibitors, and ICI plus CTLA-4 inhibitor therapy. **Results:** A total of 733 HLH-related reports associated with ICIs were identified. The median age was 65 years (range 1–92), and 54.9% of patients were male. Lung cancer (34.4%) and melanoma (16.0%) were the most frequently reported malignancies. ICI monotherapy accounted for 34.7% of cases, while combination regimens included ICI plus chemotherapy (31.6%), ICI plus targeted therapy (17.8%), and ICI plus CTLA-4 inhibitors (15.9%). All cases were classified as serious adverse events; hospitalization occurred in 69.2% and death in 25.1%. Compared with monotherapy, combination regimens showed higher reporting odds of HLH, with the strongest signal for ICI plus targeted therapy (ROR 2.17, 95% CI 1.72–2.73). PD-1 inhibitors demonstrated higher reporting odds than PD-L1 inhibitors (ROR 1.86, 95% CI 1.41–2.46). **Conclusions:** This large real-world pharmacovigilance analysis demonstrates differential HLH reporting patterns across ICI classes and treatment strategies. Higher reporting odds with combination regimens and PD-1 inhibitors highlight the need for heightened clinical vigilance, particularly in combination treatment settings.

## Simple Summary

Hemophagocytic lymphohistiocytosis (HLH) is a rare but life-threatening hyperinflammatory syndrome that has been increasingly reported in association with immune checkpoint inhibitors (ICIs). However, comparative real-world data across different ICI classes and treatment strategies remain limited. Using the U.S. Food and Drug Administration Adverse Event Reporting System (FAERS), we evaluated reporting patterns of HLH associated with ICIs. Our findings demonstrate higher reporting odds with combination regimens and PD-1 inhibitors compared with PD-L1 inhibitors. These results highlight the importance of heightened clinical vigilance, particularly in combination treatment settings.

## 1. Introduction

Hemophagocytic lymphohistiocytosis (HLH) is a rare but potentially fatal hyperinflammatory syndrome characterized by uncontrolled activation of cytotoxic T lymphocytes and macrophages, resulting in excessive cytokine release, multiorgan dysfunction, and high mortality if not promptly recognized and treated [1,2]. Although HLH has traditionally been described in pediatric populations and in association with infections, autoimmune diseases, or hematologic malignancies, its occurrence in adults has been increasingly recognized, particularly in the context of immune-modulating therapies [3].

Immune checkpoint inhibitors (ICIs) have transformed the treatment landscape of multiple solid tumors by enhancing antitumor immune responses through blockade of inhibitory immune pathways, including programmed death-1 (PD-1), programmed death-ligand 1 (PD-L1), and cytotoxic T-lymphocyte–associated antigen 4 (CTLA-4). While these agents have improved survival outcomes across diverse malignancies, they are also associated with a distinct spectrum of immune-related adverse events (irAEs), including hematologic toxicities. Among these, hemophagocytic lymphohistiocytosis (HLH) represents one of the most severe and life-threatening manifestations [4,5,6].

Immune checkpoint inhibitor–related hemophagocytic lymphohistiocytosis (irHLH) is considered a rare immune-related adverse event. Published retrospective studies and case series have reported an estimated incidence ranging from approximately 0.03% to 0.4%, with reported fatality rates of up to 50% in selected cohorts [7,8,9]. These estimates should be interpreted cautiously, as they are derived from limited retrospective studies and case reports that are subject to selection bias and may not be representative of the overall population receiving immune checkpoint inhibitors. Because the current evidence is largely based on individual case reports and small retrospective series, reliable comparative data across different ICI classes and treatment strategies remain limited. Large-scale pharmacovigilance databases such as the U.S. Food and Drug Administration Adverse Event Reporting System (FAERS) provide an opportunity to evaluate rare but serious toxicities in real-world clinical practice [10,11]. In this study, we conducted a comprehensive FAERS-based pharmacovigilance analysis to characterize HLH reporting patterns associated with ICIs and to compare disproportionality signals across ICI classes and treatment strategies.

## 2. Materials and Methods

### 2.1. Data Source and Study Design

A retrospective pharmacovigilance study was conducted using data from the publicly available FAERS database. Adverse event reports submitted between January 2013 and October 2025 were retrieved for evaluation. The FAERS database was searched for reports involving immune checkpoint inhibitors approved by the FDA within the study period. The ICIs analyzed comprised PD-1 inhibitors (cemiplimab, dostarlimab, nivolumab, pembrolizumab, sintilimab, tislelizumab, and toripalimab), PD-L1 inhibitors (atezolizumab, durvalumab, and avelumab), and CTLA-4 inhibitors (ipilimumab and tremelimumab). Reports of ICIs administered either as monotherapy or in combination with chemotherapy, targeted therapy, or CTLA-4 inhibitors were included in the analysis. FAERS data were accessed through the FAERS Public Dashboard using standardized queries based on the generic names of immune checkpoint inhibitors and MedDRA Preferred Terms related to hemophagocytic lymphohistiocytosis. Reports were retrieved at the case level, and treatment strategies were determined according to the drugs listed within each FAERS report to ensure consistent classification of monotherapy and combination regimens.

### 2.2. Case Identification and MedDRA Coding

HLH-related cases were identified using a conservative case definition based on core Medical Dictionary for Regulatory Activities (MedDRA) Preferred Terms (PTs), including hemophagocytic lymphohistiocytosis, secondary hemophagocytic lymphohistiocytosis, and macrophage activation syndrome. Broader MedDRA hierarchies or standardized MedDRA queries were not applied to avoid inclusion of non-specific inflammatory conditions and to increase diagnostic specificity, given the complexity of HLH diagnosis in spontaneous reporting databases. Additional inflammatory and hematologic MedDRA terms were reviewed to support comprehensive case ascertainment; however, these terms were not used as independent case-defining criteria [12]. Broader case definitions were considered but not adopted as primary criteria due to concerns regarding reduced specificity, and sensitivity analyses using broader definitions were not performed. This strategy was implemented to optimize diagnostic specificity and minimize misclassification within the spontaneous reporting framework. Because FAERS reports lack detailed clinical information, confirmation of HLH according to established diagnostic criteria (e.g., HLH-2004) was not possible, and cases were identified based on MedDRA Preferred Terms, consistent with standard pharmacovigilance methodology. The MedDRA Preferred Terms applied for the identification of HLH-related cases in FAERS are summarized in Supplementary Table S1.

A total of **233,457** adverse event reports related to immune checkpoint inhibitors (ICIs) were retrieved from the FAERS database. Duplicate records (n = 10,255) were identified and removed using FAERS case identification numbers. When multiple reports shared the same case identification number, reports were reviewed for consistency in key variables, including drug names, adverse event terms, patient demographics, and reporting dates, and only the most complete and most recent report was retained. Because FAERS may contain multiple reports for the same clinical case submitted by different reporters, these variables were further reviewed to reduce the likelihood of duplicate case inclusion. After duplicate removal, **233,202** unique ICI-related adverse event reports remained.

**Figure 1:**
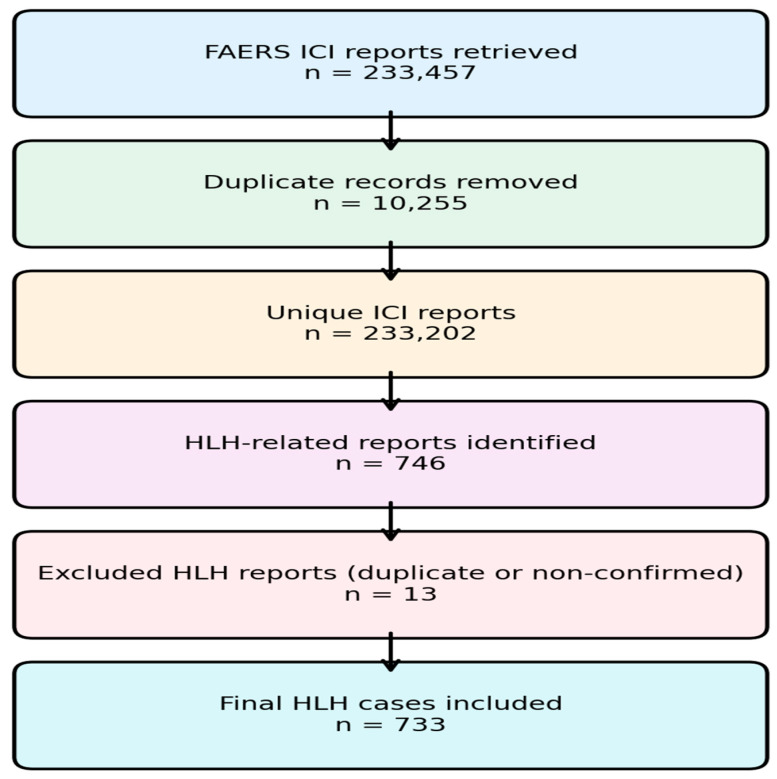
Flow diagram of case selection for hemophagocytic lymphohistiocytosis (HLH) in the FAERS database. Adverse event reports related to ICIs were retrieved from the FAERS. Duplicate records were removed, HLH-related reports were identified using MedDRA Preferred Terms, and duplicate HLH reports were excluded. The final analytical cohort consisted of 733 unique HLH cases.

Among these, 746 reports describing HLH-related adverse events were identified. Following the exclusion of duplicate HLH records (n = 13), a total of 733 unique HLH cases were included in the final analysis. HLH cases were stratified according to treatment strategy, including ICI monotherapy, ICI plus chemotherapy, ICI plus targeted therapy, and ICI plus CTLA-4 inhibitor combinations (Figure 1). Treatment strategies were determined based on the list of drugs reported in each FAERS case, and combination therapy was defined when an immune checkpoint inhibitor was reported together with chemotherapy, targeted therapy, or CTLA-4 inhibitors within the same report.

### 2.3. Statistical Analysis

Disproportionality analyses were conducted to evaluate reporting associations between ICIs and HLH. Reporting odds ratios (RORs) with corresponding 95% confidence intervals (Cis) were calculated using 2 × 2 contingency tables, in which cases were defined as HLH reports and non-cases as all other adverse event reports associated with immune checkpoint inhibitors within the FAERS database. Thus, the analysis was restricted to ICI-related reports rather than using the entire FAERS database as the comparator. For treatment strategy analyses, ICI monotherapy was used as the reference category. Additional restricted analyses compared PD-1 inhibitors, PD-L1 inhibitors, and ICI plus CTLA-4 inhibitor therapy to evaluate differences in reporting patterns across ICI classes. A signal of disproportionate reporting was considered present when the lower limit of the 95% CI exceeded 1.0. Descriptive statistics were used to summarize report characteristics and the distribution of HLH-related adverse events. Seriousness outcomes were defined according to the FAERS seriousness criteria, including hospitalization, life-threatening events, and death, as reported in each case record. All analyses were descriptive and exploratory in nature and were designed to characterize reporting patterns rather than to establish causal associations. Statistical analyses were performed using IBM SPSS Statistics for Windows, version 29. (IBM Corp., Armonk, NY, USA).

## 3. Results

### 3.1. Patient Characteristics

A total of 733 HLH-related adverse event reports associated with immune checkpoint inhibitors were identified in the FAERS database. The median patient age was 65 years (range 1–92), and 37.9% of patients were aged 65 years or older. Male patients accounted for 54.9% of reports, whereas female patients represented 37.2%; sex was not specified for a small proportion of cases. Lung cancer was the most frequently reported underlying malignancy (34.4%), followed by melanoma (16.0%), breast cancer (7.8%), renal cell carcinoma (6.8%), and hepatocellular carcinoma (6.1%). A heterogeneous group of other solid tumors comprised the remaining cases. Reports were submitted between 2013 and 2025, with 46.5% recorded during 2024–2025, 30.8% during 2021–2023, 19.5% during 2018–2020, and 2.6% before 2018; the reporting period was unknown in 0.5% of cases. Healthcare professionals accounted for 97.4% of reports and consumers for 2.3%, and reporter type was not specified in 0.3% of reports. Patient demographics, reporting characteristics, and clinical outcomes are summarized in Table 1.

### 3.2. Immune Checkpoint Inhibitor Treatment Patterns

ICI monotherapy accounted for 34.7% of HLH reports (n = 254). Combination regimens included ICI plus chemotherapy in 31.6% of cases (n = 231), ICI plus targeted therapy in 17.8% (n = 131), and ICI plus CTLA-4 inhibitor therapy in 15.9% (n = 117). Among individual ICIs, pembrolizumab accounted for 46.7% of reports, followed by nivolumab (17.3%) and atezolizumab (15.4%). Other agents were reported less frequently, including cemiplimab (1.5%), durvalumab (1.2%), dostarlimab (0.7%), sintilimab (0.7%), tislelizumab (0.3%), avelumab (0.1%), and toripalimab (0.1%). For combination regimens involving CTLA-4 inhibitors, nivolumab plus ipilimumab accounted for 14.7% of reports, followed by durvalumab plus tremelimumab (1.1%) and pembrolizumab plus ipilimumab (0.1%).

### 3.3. Clinical Outcomes

**Figure 2:**
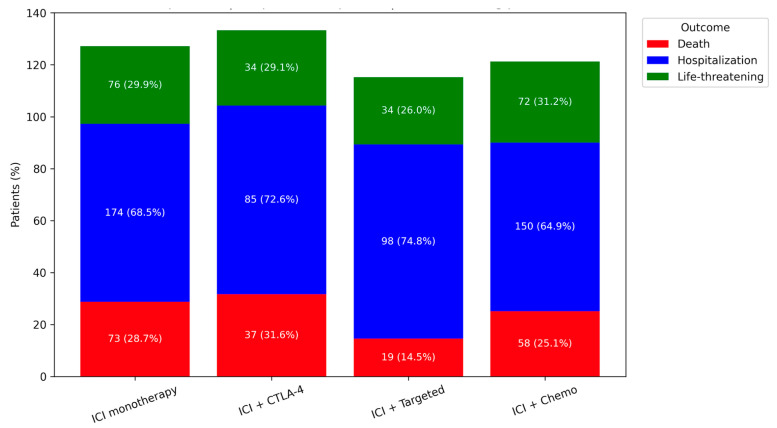
Reported clinical outcomes by immune checkpoint inhibitor (ICI) treatment strategy in the FAERS database. Footnote: Distribution of reported clinical outcomes according to treatment strategy. No statistically significant differences were observed for reported hospitalization (*p* = 0.20) or life-threatening events (*p* = 0.77). Findings are based on spontaneous reporting data and should not be interpreted as evidence of causality.

All identified HLH cases were classified as serious adverse events. Hospitalization was reported in 69.2% of cases, and life-threatening events were reported in 29.5%. Death was reported in 25.1% of cases. The frequency of reported death outcomes varied across treatment strategies (*p* = 0.007). No statistically significant differences were observed between groups in the frequency of reported hospitalization or life-threatening events. Clinical outcomes are summarized in Figure 2.

### 3.4. Disproportionality Analysis

**Figure 3:**
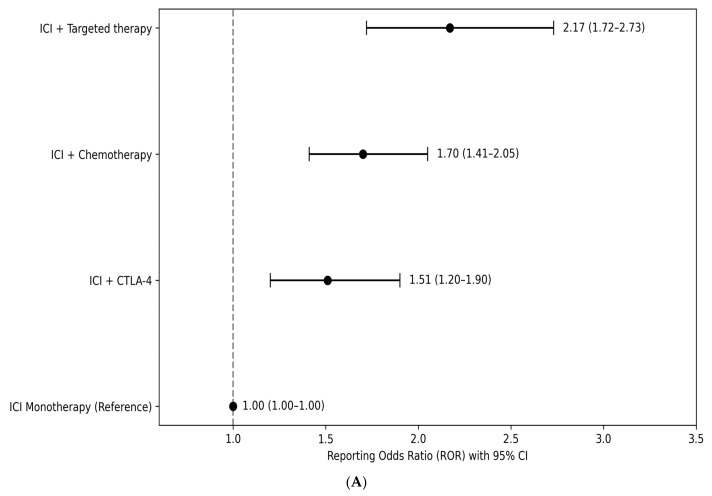

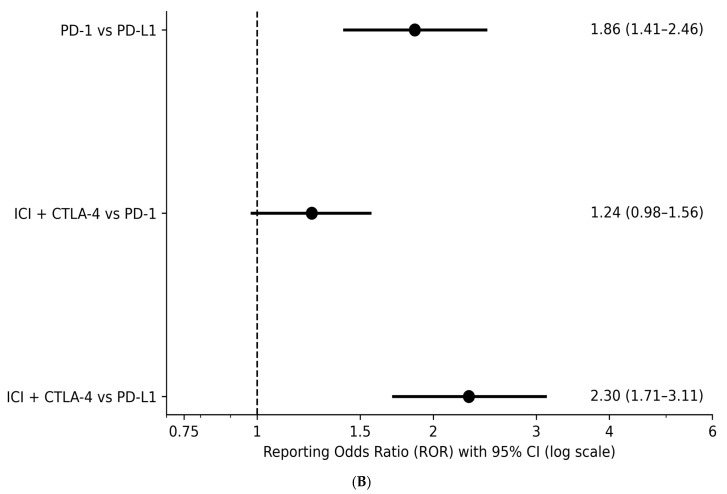
Forest plots of reporting odds ratios for hemophagocytic lymphohistiocytosis associated with immune checkpoint inhibitor treatment strategies and drug classes in the FAERS database. (**A**) Forest plot of reporting odds ratios (RORs) for HLH comparing immune checkpoint inhibitor combination regimens (ICI plus targeted therapy, ICI plus chemotherapy, and ICI plus CTLA-4 inhibitor therapy) with ICI monotherapy (reference). Footnote: Forest plot showing reporting odds ratios (RORs) with 95% confidence intervals for HLH associated with different ICI-based treatment strategies compared with ICI monotherapy. Numeric ROR values with confidence intervals are displayed next to each estimate. The vertical dashed line indicates the reference value (ROR = 1). Results are based on spontaneous reports in FAERS and represent disproportionality signals rather than causal relationships. (**B**) Forest plot of reporting odds ratios (RORs) for HLH comparing programmed death-1 (PD-1) inhibitors with programmed death-ligand 1 (PD-L1) inhibitors and comparing ICI plus CTLA-4 inhibitor therapy with PD-1 and PD-L1 inhibitors in restricted analyses. Footnote: Forest plot showing reporting odds ratios (RORs) with 95% confidence intervals for HLH in restricted analyses. Numeric ROR values with confidence intervals are displayed next to each estimate. The vertical dashed line indicates the reference value (ROR = 1). Findings are based on spontaneous reports in FAERS and represent disproportionality signals rather than causal relationships.

Compared with ICI monotherapy, combination regimens were associated with higher reporting odds of HLH. The strongest disproportionality signal was observed for ICI plus targeted therapy (ROR 2.17, 95% CI 1.72–2.73), followed by ICI plus chemotherapy (ROR 1.70, 95% CI 1.41–2.05) and ICI plus CTLA-4 inhibitor therapy (ROR 1.51, 95% CI 1.20–1.90). In restricted analyses limited to patients receiving ICI monotherapy and those treated with ICI plus CTLA-4 inhibitors, PD-1 inhibitors showed higher reporting odds of HLH compared with PD-L1 inhibitors (ROR 1.86, 95% CI 1.41–2.46). Within this restricted cohort, ICI plus CTLA-4 inhibitor therapy was associated with higher reporting odds compared with PD-L1 inhibitors (ROR 2.30, 95% CI 1.71–3.11), whereas no statistically significant difference was observed when compared with PD-1 inhibitors (ROR 1.24, 95% CI 0.98–1.56). Reporting odds ratios for HLH according to immune checkpoint inhibitor treatment strategy and drug class are shown in Figure 3. These findings represent disproportionality signals derived from spontaneous reports and should not be interpreted as estimates of incidence or causal risk.

## 4. Discussion

In this large-scale pharmacovigilance study based on the FAERS database, we evaluated hemophagocytic lymphohistiocytosis (HLH) as a rare but severe immune-related adverse event reported in association with immune checkpoint inhibitor (ICI) therapy. By analyzing more than 233,000 unique ICI-related adverse event reports and identifying 733 HLH cases, this study represents one of the most comprehensive real-world pharmacovigilance assessments of ICI-associated HLH to date. Rather than estimating incidence or absolute risk, our analysis was designed to characterize reporting patterns and disproportionality signals across ICI classes and treatment strategies. These findings should be interpreted cautiously, as differences in reporting patterns may reflect variations in drug utilization, prescribing practices, and reporting behavior rather than true biological differences in risk.

Previous pharmacovigilance studies and case-based reviews have identified hemophagocytic lymphohistiocytosis (HLH) as a rare but severe immune-related adverse event associated with immune checkpoint inhibitors; however, comparative analyses across drug classes and treatment strategies remain limited. Noseda et al. described the clinical features and outcomes of ICI-associated HLH using the WHO global database but did not perform stratified disproportionality analyses [7]. Rajapakse and Andanamala summarized published case reports and small case series, providing descriptive clinical insights but without comparative evaluation [13]. Diaz et al. later confirmed a disproportionate reporting signal for HLH in a pharmacovigilance study combining global database reports with literature cases, although detailed stratification by ICI class and treatment strategy was not performed [14]. In contrast, the present FAERS-based analysis includes a substantially larger number of HLH reports and allows systematic comparisons across ICI classes and treatment strategies, including direct evaluation of PD-1 versus PD-L1 inhibitors and combination regimens. A summary of previous studies and the current findings is provided in Table 2.

The higher reporting odds of HLH observed with combination treatment strategies may reflect synergistic immune activation rather than the effect of a single agent. Immune checkpoint inhibitors enhance T-cell–mediated antitumor immunity by releasing inhibitory immune pathways, whereas concomitant targeted therapies or CTLA-4 blockade may further augment immune stimulation through complementary mechanisms. Targeted therapies may modulate the tumor microenvironment by increasing antigen presentation, altering cytokine profiles, and promoting immunogenic cell death, thereby potentially lowering the threshold for dysregulated immune activation when combined with immune checkpoint blockade [15]. Similarly, the increased reporting odds observed with ICI plus CTLA-4 inhibitor therapy are biologically plausible given the broader and more proximal immune activation associated with CTLA-4 blockade, which enhances early T-cell priming and expansion in secondary lymphoid organs and may result in more systemic immune activation [16]. The disproportionate reporting of HLH observed with PD-1 inhibitors relative to PD-L1 inhibitors may also be related to differences in the scope and intensity of immune modulation mediated by these agents. PD-1 blockade targets inhibitory receptors on activated T cells and may result in broader immune activation, whereas PD-L1 inhibition blocks ligand–receptor interactions at the tumor or antigen-presenting cell interface, potentially preserving partial inhibitory signaling. Experimental and translational studies have suggested that PD-1 blockade may be associated with greater expansion of effector T cells and higher cytokine production compared with PD-L1 inhibition. These mechanistic differences may be relevant for HLH, a systemic hyperinflammatory syndrome characterized by excessive cytokine release and uncontrolled macrophage and lymphocyte activation [17,18,19]. However, PD-1 inhibitors are prescribed more widely across multiple malignancies compared with PD-L1 inhibitors, which may increase the number of reports captured in pharmacovigilance databases. In addition, the predominance of pembrolizumab reports in the present dataset likely reflects its earlier approval, broader utilization, and wider range of clinical indications compared with other ICIs, rather than a true difference in HLH risk. Because FAERS does not provide reliable denominator data on drug exposure, the observed differences in reporting odds should be interpreted cautiously and may reflect variations in drug utilization rather than true biological differences in HLH risk. Therefore, the stronger disproportionality signals observed with combination regimens and PD-1 inhibitors should be interpreted as differences in reporting patterns rather than evidence of a causal increase in HLH risk. In FAERS, combination therapy was defined based on co-reporting within the same case record, and a reliable distinction between concomitant and sequential administration is not possible. Furthermore, confounding factors such as underlying disease severity, cytotoxic chemotherapy, or targeted therapies cannot be fully controlled, and the observed associations should therefore be interpreted as reporting signals rather than causal effects.

The management of immune checkpoint inhibitor–associated hemophagocytic lymphohistiocytosis remains non-standardized and is largely based on evidence from case reports and small case series. High-dose corticosteroids constitute the cornerstone of initial therapy, whereas cytokine-directed strategies, particularly interleukin-6 inhibition, have emerged as potential therapeutic options in steroid-refractory cases. Several published reports suggest that targeting key inflammatory mediators may be associated with biochemical and clinical improvement in selected patients [9,20,21,22]. Hemophagocytic lymphohistiocytosis is a severe and potentially fatal syndrome, with reported mortality rates exceeding 40–50% in some clinical settings [7,8,9]. The mortality observed in the present analysis should therefore be interpreted cautiously, as spontaneous reporting databases may preferentially include more severe cases and do not allow reliable estimation of baseline risk. However, the available evidence is derived from non-comparative studies, and substantial heterogeneity exists in reported treatment approaches across published cases. Importantly, the present pharmacovigilance analysis does not allow evaluation of specific therapeutic interventions or treatment strategies, thereby precluding any systematic assessment of management approaches for ICI-associated HLH.

This study has several limitations inherent to the use of the FAERS database. As a spontaneous and voluntary reporting system, FAERS is subject to substantial underreporting, selective reporting, and heterogeneity in data quality and completeness. FAERS analyses are also limited by the absence of reliable denominator data, confounding by indication, and potential temporal reporting bias, which may influence the observed reporting patterns, particularly with increased reporting in recent years [23,24,25]. Furthermore, FAERS does not allow adjustment for important confounding factors such as differences in underlying malignancies, disease stage, prior therapies, or concomitant medications. Disproportionality analyses based on reporting odds ratios cannot fully account for differences in reporting volume, publicity bias, or variations in clinical indications across drug classes and therefore should not be interpreted as direct comparisons of risk. Similarly, comparisons between ICI classes cannot be normalized for differences in treated patient populations or time on the market, as FAERS does not provide reliable exposure data. Individual reports often lack important clinical details, including comorbidities, drug dosing, timing of adverse event onset, and diagnostic confirmation of HLH, which may lead to misclassification of both drug exposure and reported outcomes. Because FAERS reports do not provide sufficient clinical detail to apply established diagnostic criteria such as HLH-2004, case identification relied on MedDRA Preferred Terms, which may introduce diagnostic misclassification. Consequently, FAERS-based disproportionality analyses are inherently hypothesis-generating and cannot establish causal relationships. In addition, detailed sensitivity analyses according to reporting year, underlying malignancy, or concomitant medications were not feasible because of incomplete clinical information. Seriousness outcomes recorded in FAERS may reflect the overall clinical condition of the patient rather than complications directly attributable to HLH, and information on HLH management and clinical outcomes is generally unavailable, limiting evaluation of treatment approaches. Pharmacovigilance data may also be influenced by regional differences in regulatory requirements, reporting practices, and reporting culture, and therefore FAERS-based analyses may not fully reflect global reporting patterns. Despite these limitations, FAERS remains a valuable resource for identifying rare and potentially serious toxicities that may not be detected in clinical trials.

## 5. Conclusions

We present a large-scale pharmacovigilance analysis using FAERS data to characterize reporting patterns and clinical severity of immune checkpoint inhibitor–associated hemophagocytic lymphohistiocytosis. Our findings are consistent with the existing literature, confirming that HLH is a rare but severe immune-related adverse event, predominantly associated with PD-1 inhibitors and more frequently reported in the setting of combination treatment strategies. The uniformly serious nature of reported cases, reflected by high rates of hospitalization and mortality, highlights the clinical relevance of this complication. Although spontaneous reporting data do not allow estimation of incidence or establishment of causality, the observed disproportionality patterns are consistent with the hypothesis that cumulative immune activation plays an important role in HLH pathogenesis. Further prospective and mechanistic studies are warranted to improve risk stratification, refine monitoring strategies, and identify predictive biomarkers, thereby facilitating earlier recognition and safer use of immune checkpoint inhibitors in patients at risk for hyperinflammatory immune-mediated toxicities.

## Figures and Tables

**Table 1:** Demographic and reporting characteristics of HLH with ICIs in the FAERS database.

Variable			n = 733 (%)
Age		Median	65 (min 1–max 92)
		≥65	278 (37.9)
Gender		Female	122 (37.2)
		Male	180 (54.9)
		Not specified	26 (7.9)
Primary Malignancy		Lung cancer	252 (34.4)
		Melanoma	117 (16.0)
		Renal cell carcinoma	50 (6.8)
		Breast cancer	57 (7.8)
		Colorectal cancer	8 (1.1)
		Hepatocelular carcinoma	45 (6.1)
		Head and neck cancer	15 (2.0)
		Urothelial cancer	7 (1.0)
		Gastric cancer	19 (1.8)
		Prostate cancer	13 (0.9)
		Lymphoma	9 (1.2)
		Esophageal cancer	9 (1.2)
		Ovarian cancer	5 (0.5)
		Other/not specified	127 (17.3)
Treatment		ICI monotherapy	254 (34.7)
		ICI + CTLA-4 inhibitor	117 (15.9)
		ICI + Targeted therapy	131 (17.8)
		ICI + Chemotherapy	231 (31.6)
ICI agent		Atezolizumab	113(15.4)
		Avelumab	1 (0.1)
		Cemiplimab	11 (1.5)
		Dostarlimab	5 (0.7)
		Durvalumab	9 (1.2)
		Nivolumab	127 (17.3)
		Pembrolizumab	342 (46.7)
		Sintilimab	5 (0.7)
		Tislelizumab	2 (0.3)
		Toripalimab	1 (0.1)
ICI + CTLA 4 inhibitor		Durvalumab + Tremelimumab	8 (1.1)
		Nivolumab + Ipilimumab	108 (14.7)
		Pembrolizumab + Ipilimumab	1 (0.1)
Reporting Period		<2018	19 (2.6)
		2018–2020	143 (19.5)
		2021–2023	226 (30.8)
		2024–2025	341 (46.5)
		Unknown	4 (0.5)
Reporter Type		Healthcare worker	714 (97.4)
		Consumer	17 (2.3)
		Unknown	2 (0.3)
Outcomes	Serious outcomes		733 (100)
	Hospitalization		507 (69.2)
	Life threatening		216 (29.5)
	Death		187 (25.1)

**Table 2:** Comparison of pharmacovigilance and case-based studies evaluating immune checkpoint inhibitor–associated hemophagocytic lymphohistiocytosis.

Feature	Noseda et al., [7]	Rajapakse & Andanamala, [13]	Diaz et al., [14]	Present Study
Data source	WHO VigiBase	Published case reports/series	WHO VigiBase + literature	FAERS (U.S. FDA Adverse Event Reporting System)
Study design	Pharmacovigilance analysis	Systematic case review	Pharmacovigilance disproportionality analysis	Pharmacovigilance disproportionality analysis
Number of HLH cases	38	22	190	733
ICI classes evaluated	PD-1, PD-L1, CTLA-4	PD-1, PD-L1, CTLA-4	PD-1, PD-L1, CTLA-4	PD-1, PD-L1, CTLA-4
Most frequent agents	Nivolumab n = 14 (37%) Pembrolizumab n = 7 (18%) ipilimumab monotherapy n = 7 (18%) ipilimumab and nivolumab combination therapy n = 5 (13%)	Pembrolizumab n = 13 (59%) Nivolumab monotherapy n = 2 (10%) Nivolumab + ipilimumab n = 7 (31%)	Pembrolizumab n = 58 (30%) Nivolumab n = 21 (11%) Nivolumab + ipilimumab n = 65 (34%)	Pembrolizumab n = 342 (46.7%) Nivolumab n = 127 (17.3%) Nivolumab + ipilimumab n = 108 (14.6%)
Treatment strategy stratification	No	Descriptive only	Limited	Systematic
Main finding	First large description of ICI-associated HLH and its clinical features	Clinical presentation and treatment patterns of ICI-associated HLH from reported cases	Confirmed disproportionate reporting signal for HLH with ICIs	Differential reporting across ICI classes and higher reporting with combination regimens
Clinical severity	All cases serious; 42% hospitalized and 26% fatal	All cases required inpatient care; ~14% fatal	All cases serious; 15% fatal	All cases serious; 25% fatal
Key contribution	First large-scale pharmacovigilance analysis of ICI-associated HLH	Clinical characterization and management of ICI-associated HLH from published case reports	Largest pharmacovigilance series confirming HLH signal with ICIs	Largest comparative and stratified pharmacovigilance analysis of ICI-associated HLH
Main limitation	Limited sample size and lack of stratified analyses	Small sample size and descriptive, non-comparative design	Limited stratification by ICI class and treatment strategy	Underreporting and reporting bias inherent to spontaneous reporting databases; lack of denominator data

Footnotes: HLH, hemophagocytic lymphohistiocytosis; ICI, immune checkpoint inhibitor; FAERS, U.S. Food and Drug Administration Adverse Event Reporting System; WHO, World Health Organization. Percentages refer to the proportion of HLH cases within each study.

## Data Availability

The datasets generated and/or analyzed during the current study are available from the corresponding author.

## Supplementary Materials

The following supporting information can be downloaded at: https://www.mdpi.com/article/10.3390/cancers18071164/s1, Table S1 . MedDRA Preferred Terms (PTs) used to identify HLH-related cases in FAERS.

## Abbreviations

CI	Confidence Interval
CTLA-4	Cytotoxic T-Lymphocyte–Associated Antigen 4
FAERS	U.S. Food and Drug Administration Adverse Event Reporting System
FDA	U.S. Food and Drug Administration
HLH	Hemophagocytic Lymphohistiocytosis
ICI	Immune Checkpoint Inhibitor
irAE	Immune-Related Adverse Event
irHLH	Immune Checkpoint Inhibitor–Related Hemophagocytic Lymphohistiocytosis
MedDRA	Medical Dictionary for Regulatory Activities
PD-1	Programmed Death-1
PD-L1	Programmed Death-Ligand 1
PT	Preferred Term
ROR	Reporting Odds Ratio
WHO	World Health Organization
