# Supplementary material for: Hemophagocytic Lymphohistiocytosis Associated with Immune Checkpoint Inhibitors: A Pharmacovigilance Analysis of Spontaneous Reports
Source: Cancers (Basel). 2026 Apr 4;18(7):1164. doi: 10.3390/cancers18071164 (PMC13072010; doi:10.3390/cancers18071164)
Supplement: Supplementary file 1 [file cancers-18-01164-s001.zip › cancers-4210848-supplementary.pdf]

Supplementary Table S1. MedDRA Preferred Terms (PTs) used to identify HLH-related cases in FAERS

Category	MedDRA Preferred Term (PT)
Core HLH terms	Hemophagocytic lymphohistiocytosis
	Secondary hemophagocytic lymphohistiocytosis
	Macrophage activation syndrome
Related inflammatory syndromes	Cytokine release syndrome
	Hyperinflammatory syndrome
	Systemic inflammatory response syndrome
Hematologic manifestations	Pancytopenia
	Bone marrow failure
	Febrile neutropenia
Laboratory findings	Hyperferritinaemia
Severe systemic outcomes	Multiple organ dysfunction syndrome
	Sepsis

Explanation:

HLH-related cases were primarily identified using the core MedDRA Preferred Terms “Hemophagocytic lymphohistiocytosis,” “Secondary hemophagocytic lymphohistiocytosis,” and “Macrophage activation syndrome.” Additional clinically related PTs were reviewed to capture hyperinflammatory presentations potentially consistent with HLH. These supplementary terms were not used as standalone case-defining criteria but served to support comprehensive case identification.
